# Importance of the National Early Warning Score (NEWS) at the time of discharge from the intensive care unit

**DOI:** 10.3906/sag-1906-78

**Published:** 2020-08-26

**Authors:** Cihangir DOĞU, Güvenç DOĞAN, Selçuk KAYIR, Özgür YAĞAN

**Affiliations:** 1 Department of Intensive Care, Republic of Turkey Ministry of Health Ankara City Hospital, Ankara Turkey; 2 Department of Anesthesiology and Reanimation, Faculty of Medicine, Hitit University, Çorum Turkey

**Keywords:** Intensive care, early warning score, patient readmission, patient discharge COPD

## Abstract

**Backround/aim:**

To identify, at an early stage of intensive care, patients who will require readmission to the intensive care unit (ICU) based on their National Early Warning Score (NEWS-d) at discharge.

**Materials and methods:**

Overall, 536 patients aged >18 years who stayed at a tertiary ICU for >24 h were included. Patients who readmitted and not readmitted to the intensive care within 48 h after discharge were compared.

**Results:**

Mean patient age was 64.26 ± 18.50 years and 252 (44.7%) patients were male. Mean Acute Physiology and Chronic Health Evaluation II (APACHE II) score was 21.86 ± 8.74; mean NEWS-d was 4.48 ± 2.53. Forty-nine (9.1%) were readmitted to ICU. The reasons for initial admission, age, and NEWS-d vvalues were significantly different between the 2 groups. The NEWS-d values of the readmitted group were significantly higher (9.16 ± 1.05) than nonreadmitted group (4.01 ± 2.13). Based on receiver operation curve analysis, sensitivity and specificity were 98% and 95%, respectively, considering a NEWS-d cut-off value of 7.5 as the limit value for estimating readmission.

**Conclusion:**

A NEWS-d value of >7.5 demonstrated high sensitivity and specificity in identifying the risk of readmission for patients being discharged from ICU.

## 1.Introduction

The discharge of critical care patients from the intensive care unit (ICU) to the general ward without delay helps to reduce costs and enables the reasonable use of the valuable ICU beds [1]. However, transferring patients who require care following their stay in ICU before they are ready for the general ward increases the probability of their readmission into ICU [2]. Reportedly, readmitted patients have higher mortality and length of stay at the hospital and ICU [3,4]. Moreover, readmission rates are currently being used as a quality criterion for intensive care services [5]. Therefore, studies are being conducted for the development of various estimation tools that would prevent early discharge from ICUs [6].

The National Early Warning Score (NEWS) is a scoring system that is recommended by the Royal College of Physicians (RCP), London, UK and it is designed for the early diagnosis of deterioration in the physiological parameters of patients [7]. It consists of the evaluation of 6 vital signs as well as the oxygen saturation of the patient. High NEWS scores are associated with more severe disease and poorer outcomes [8,9]. NEWS scores as well as admission to the emergency services and inpatient wards were evaluated, and it was shown that their predictive levels was high [10]. Discharge of the patients from ICU typically occurs following the resolution of a life-threatening condition and upon the discretion of the intensive care physician. Discharge criteria tend to vary. NEWS score has been reported to be predictive in discharges from primary ICUs and readmissions to ICU [11]. However, a similar study has not yet been conducted for tertiary ICUs. There is no actively used scoring system accepted at the discharge from the intensive care unit.

In the present study, our primary purpose was to identify patients requiring readmission to ICU at an early stage by examining NEWS-d at the time of discharge from ICU. Our secondary purpose was to determine a NEWS-d cutoff for identifying patients at a risk of readmission.

## 2. Materials and methods

This prospective cohort study was planned at a single center and was conducted in compliance with the Declaration of Helsinki. Following the ethics committee approval of Hitit University (decision number: 2018-06), it was registered at ClinicalTrials.gov (registration number NCT03626961). Informed consent forms were obtained from the patients and/or their relatives.

Patient characteristics: Between January 2018 and January 2019 discharged patients aged >18 years, who had a longer length of stay more than 24 h to tertiary ICU for medical and postsurgical therapy included in the study. 

Patients who were younger than 18 years old age and patients had a length of stay less than 24 h excluded from the study. Patients who died in the ICU and the patients with intoxications excluded from the study. 

Discharge: The decision of discharging the patients from ICU was made as per the discretion of the intensive care physician as well as of the physician of the ward to which the patient will be discharged. Patients were discharged into lower-level ICUs, wards, or palliative care centers. At the time of discharge all patients had hemodynamicly stable, Glasgow Coma Scale (GCS) >13, with (2 lt/min) or without suplemantal oxygen support and urine output more than 0,5 mL/kg/min. 

Readmission: Defined as a second admission within the first 48 h after discharge. Causes of readmission included were neurologic deterioration (GCS < 12), respiratory failure (oxygen support need more than 6 lt/min), and hemodynamic instability (need for vasoactive agents, mean bood pressure less than 60 mmHg). For patients with multiple admissions, only the first admissions were evaluated.

The following data and study variables were recorded: patient demographics, Acute Physiology and Chronic Health Evaluation II (APACHE II) scores, admission causes, length of stay. NEWS-d values of the patients were calculated at the time of discharge to wards. The clinical conditions of the patients during the 48 h after discharge from ICU were examined by the researchers, and readmitted patients were recorded.

The NEWS scoring: NEWS is recommended by the RCP for helping clinicians via the use of a simple scoring system for assessing the changes in the patients’ acute medical condition. Scoring is performed by evaluating the respiratory rate, oxygen saturation, oxygen support requirement, body temperature, heart rate, systolic blood pressure, and state of consciousness at the time of admission to the hospital. Patients with a score between 0–4 are considered as having low risk, those with a score of 5 or 6 are considered as having medium risk, and those with a score > 6 are considered as having a high risk. NEWS-d scores were calculated by the patient’s nurse at the time of the patient’s intensive care discharge. The NEWS-d values are presented in Table 1. 

**Table 1:** NEWS scoring.

Physiological parameters	3	2	1	0	1	2	3
Respiratory rate (/min)	8		9–11	12–20		21–24	>25
Oxygen saturation (%)	<91	92–93	94–95	>96			
Oxygen support requirement		Yes		No			
Body temperature (℃)	<35		35.1–36	36.1–38	38.1–39	39.1	
Systolic blood pressure (mmHg)	<90	91–100	101–110	111–219			>220
Heart rate (/min)	<40		41–50	51–90	91–110	111–130	>131
State of consciousness				Awake			With sound, with pain or nonresponsive

Our study was conducted in a closed ICU with 34 beds that provides tertiary intensive care services and monitors for medical and surgical intensive care patients. Routine hemodynamic monitoring (electrocardiogram, respiratory rate, systolic arterial pressure, body temperature, SPO2) and consciousness status were evaluated and recorded on admitting the patients to our ICU. 

Power analysis: Posthoc power analysis was done with G * Power 3.1.9.2 statistical software. The power of the study was found (1-β) = 0.95 when nonreadmitted group had 487 patients, readmitted group had 49 patients, assumed alpha error was 0.05 and effect size was 0.5.

### 2.2. Statistical analysis

Data analyses were conducted by using SPSS for Windows, version 22.0 (IBM Corp., Armonk, NY, United States). Kolmogorov–Smirnov test was used to determine whether the distribution of the continuous variables was normal. Levene test was used to evaluate the homogeneity of variances. Continuous data were defined as mean ± SD, and categorical data were defined with the number of cases (%).

Differences in variables showing normal distribution between two independent groups were statistically compared using the Student t-test. Categorical variables were compared using Pearson’s chi-square test or Fisher’s exact test.

First, univariate multinominal logistic regression analysis was used on the risk factors considered to be associated with readmission. Risk factors with a P value of <0.25 in the univariate logistic regression analysis were included in the multivariate logistic regression model. Wald statistics was used to determine whether each independent variable was significant on the model. Nagelkerke R2 was used to evaluate the number of independent variables that explained the dependent variables. Moreover, model adaptation of the estimations was evaluated using the Hosmer and Lemosow model adaptation test. Receiver operating curve (ROC) analysis was used to determine the cut-off points. In all statistical analyses, P < 0.05 was considered significant.

## 3. Results

**Figure 1:**
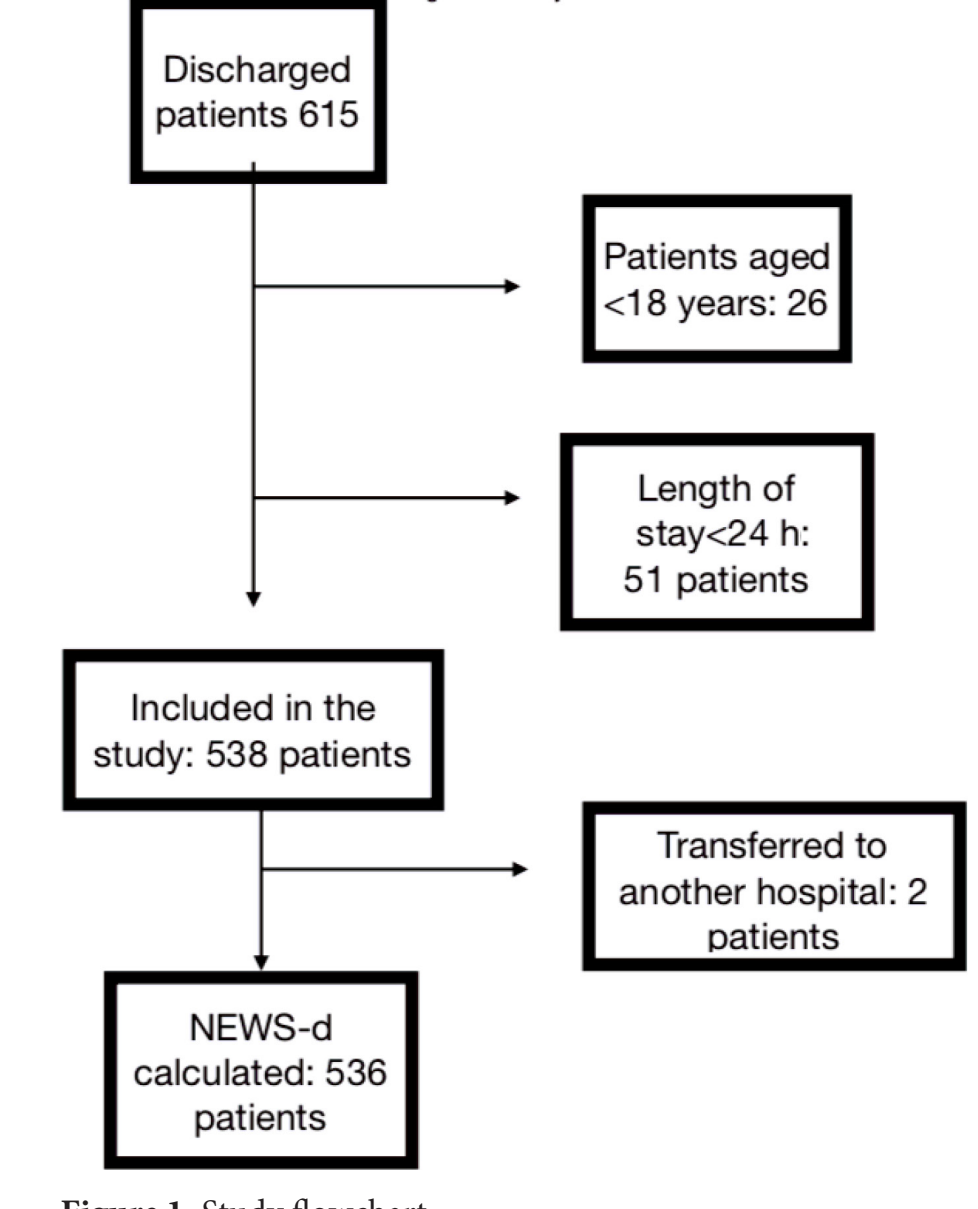
Study flowchart.

A total of 26 patients who were transferred from ICU were aged <18 years, and hence, were excluded from the study. Moreover, 51 patients who were treated at ICU for <24 h and 2 patients who were transferred to an external center were excluded from the study. Overall, 536 patients were included in the study. The flowchart is shown in Figure 1.

Mean patient age was 64.26 ± 18.50 years, and the number of male patients was 252 (44.7%). Mean APACHE II score was 21.86 ± 8.74. Mean NEWS-d was calculated as 4.48 ± 2.53. On analyzing the causes of intensive care admissions, the underlying cause was observed to be postoperative care in 97 (18.1%) patients, acute respiratory failure in 71 (13.2%) patients, and chronic obstructive pulmonary disease (COPD) in 51 (9.5%) patients.

Of these patients, 49 (9.1%) were readmitted to ICU within the first 48 h after discharge from ICU. Comparison of the readmitted patients with nonreadmitted patients revealed: it was observed that their causes for readmission to ICU, age, and NEWS-d values were significantly different. A comparison of the groups with and without readmission is presented in Table 2. The NEWS-d value was 9.16 ± 1.05 for the readmitted patients and 4.01 ± 2.13 for nonreadmitted patients.

**Table 2:** Clinical characteristics of the patients discharged from ICU (mean ± SD).

	Readmittedpatients	Nonreadmitted patients	P
Age (years)	75.63 ± 10.84	63.12 ± 18.73	<0.001ª
APACHE II	22.02 ± 8.03	21.84 ± 8.82	0.892
Length of stay (days)	9.90 ± 8.25	6.43 ± 6.64	0.006ª
NEWS	9.16 ± 1.05	4.01 ± 2.13	<0.001ª
Chronic obstructive pulmonary disease (n, %)	14 (28.6%)	37 (7.6%)	<0.001ᵇ
Respiratory failure (n, %)	12 (24.5%)	59 (12.1%)	0.025ᵇ
Cardiac arrest (n, %)	12 (24.4%)	22 (4.5%)	<0.001ᵇ
Traffic accident (n, %)	−	37 (7.6%)	0.039ᵇ
Postoperative care (n, %)	1 (2.0%)	96 (19.7%)	0.002ᵇ

a: Student t-test, b: Pearson’s chi-square test.

Univariate logistic analysis revelaed, it was observed that respiratory failure, COPD, and post cardiac arrest, as the causes of length of intensive care stay and intensive care admission, increased the risk for readmission, whereas the need for postoperative care decreased the risk of readmission. In the multivariate logistic regression analysis, only age and NEWS-d demonstrated a high power to predict readmission (Table 3).

**Table 3:** Evaluation of the causes of intensive care admission, NEWS, age, ICU length of stay in terms of readmission with regression analysis.

		Univariate logisticregression analysis				Multivariate logistic analysis		
	Wald	PR†	CI ‡	p	Wald	PR†	CI ‡	p
Age (years)	18.165	1.052	1.028–1.077	<0.001	3.840	1.052	1.000–1.107	0.049
ICU LOS☸ (days)	10.034	1.051	1.019–1.084	0.002				
NEWS	35.191	12.28	5.364– 28.144	<0.001	31.75	13.53	5.470–33.497	<0.001
COPD*	19.36	4.865	2.405–9.842	<0.001				
Respiratory failure	5.646	2.353	1.162–4.765	0.017				
Cardiac arrest	23.45	6.855	3.146–14.939	<0.001				
Postoperative care	5.886	0.085	0.012–0.622	0.015				

†: Odds ratio, ‡: Confidence interval, *: Chronic obstructive pulmonary disease, ☸: Length of stay. In the univariate logistic regression analysis, the variables of the multivariate analyses were excluded owing to P being >0.25. Cox-Snell R square: 0.373, Hosmer–Lemeshow P: 0.7998, Confidence interval: 95%.

An increase in age by one year increased the risk of readmission by 1052 times, whereas an increase in the NEWS-d value by one unit increased the risk of readmission by 13 times.

**Figure 2:**
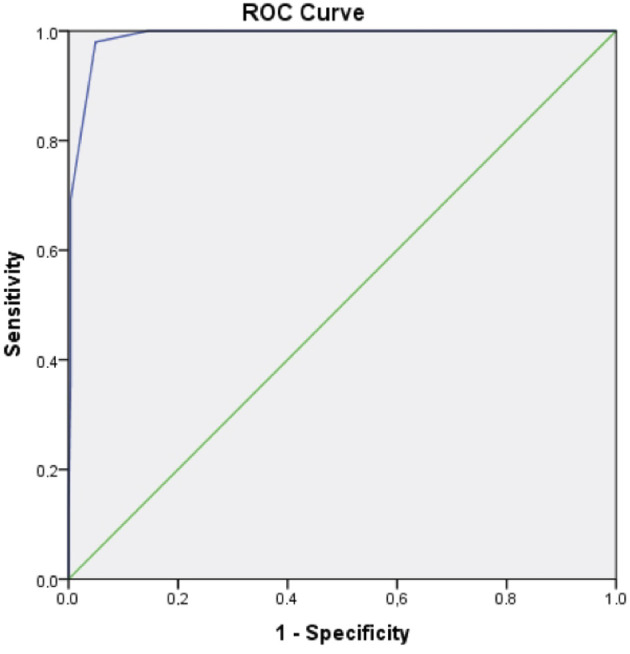
Receiver operating characteristics (ROC) curve for readmission to intensive care. For NEWS, the area under ROC is 0,989 ± 0,004 (95% CI 0.981-0.996, P < 0.001). NEWS value of 7.5 has 98% sensitivity and 95% specificity for the estimation of readmission to intensive care unit.

In the ROC analysis, sensitivity and specificity were calculated as 98% and 95%, respectively, when a NEWS-d cut-off value of 7.5 was accepted as the limit value for estimating readmission (Figure 2).

## 4. Discussion

The results of our study demonstrated that a NEWS-d value of >7.5 at the time of discharge from ICU estimated a high probability of readmission within the first 48 h after discharge. Patient age and intensive care length of stay were observed as risk factors for readmission. We determined that patients who were admitted due to respiratory failure, COPD, and postcardiac arrest exhibited a higher risk of readmission to ICU.

 NEWS is recommended by the RCP as an indicator of acute physiological deterioration for the close monitoring of patients [7] . It is a parameter that shows the risk of sudden cardiac arrest and the need for intensive care [8] and is used for admission to ICUs; additionally, it can be used for the discharge from ICUs. In their study about primary ICUs, Uppanisakorn et al. reported that readmission can be estimated for patients with a NEWS-d value of >7. In the center where the study was conducted, unplanned ICU transfers owing to high patient volume were reported, whereas the rate of readmission was reported as 14% [11]. The absence of lower-level ICUs and mandatory discharges were stated as the limitations of the study.

In their study evaluating the geriatric patient population requiring mechanical ventilatory support, Chen et al. reported that a NEWS-d value of >10 and a need for ventilatory support at the 14th day after intensive care increases mortality by 15 times. The researchers emphasized the need for ventilation monitoring of geriatric patients with high NEWS-d values receiving mechanical ventilatory support in ICU [12]. There are no studies evaluating the readmission requirements and NEWS values of patients receiving tertiary medical and surgical intensive care. In our study, we observed that the NEWS-d value calculated at the time of transfer for patients who were readmitted following their transfer from a tertiary ICU was significantly higher compared with that for patients who did not require readmission. For determining NEWS-d value, there are three parameters for respiratory sufficiency and two parameters for cardiovascular sufficiency. In case deterioration is observed in these parameters, the decision of discharge from ICU should be reevaluated. Increased respiratory and heart rates and emerging oxygen support requirement during transfer from the intensive care have previously been reported as independent variables for readmission [13]. We believe that it is possible to predict the patients who may require readmittance using NEWS-d values, which are easy to calculate and practically computable.

In determining cardiac readmissions, the area under the ROC (AUC) for NEWS-d was calculated as 0.989, and when a cut-off value of 7.5 was accepted as the limit value, sensitivity and specificity were calculated as 98% and 95%, respectively. We believe that a NEWS-d value of >7.5 that is calculated at the time of discharge from ICU can identify a risk of readmission. Moreover, in the multivariate logistic regression analyses, we observed that an increase in the NEWS-d value by one unit increases the risk of readmission by 13.5 times. An increase in the risk of readmission in parallel with the deterioration of physiological parameters is consistent with findings reported in the literature.

A previous study has shown the need for intensive care monitoring when the NEWS-d value of patients is ≥6 [8]. In our study, this value was found to be 4.01 ± 2.13 in the nonreadmitted group and 9.16 ± 1.05 in the readmitted group. Because intensive care monitoring tends to continue in patients who are transferred from tertiary ICUs to lower-level wards, patients with higher NEWS-d values were easily transferred to lower-level ICUs. It was determined that patients who are transferred to palliative care centers could be discharged with higher NEWS-d values owing to their already poor chronic health conditions.

The rate of readmission in our study was found to be 9.1%. In their study analyzing 2852 patients receiving surgical intensive care, Kaben et al. reported the rate of readmission as 13.4%. Moreover, they reported that readmitted patients had more severe sepsis and more comorbidities at their first admission to intensive care [3]. In their multicenter retrospective study that included 229,000 patients, Kramer et al. observed that the rate of readmission was 6.1%. However, that study could not evaluate the readmissions of patients who were transferred to other centers, who were discharged home, and who were transferred to postacute care centers. Moreover, they reported that patients who were admitted to other centers were not included in their study as readmissions and that they found the rate of readmission to be low [14] In a study by Russell, the rate of readmission was reported as 10.5% [15], whereas Araujo et al. reported this rate as 9.3% in the trauma ICU and as 13.7% in the mixed ICU [16] . We found that the rate of readmission observed in our mixed ICU was consistent with those observed in other studies. We believe that the prospective nature of our study and the exclusion of the patients who were transferred to other centers have provided more accurate results.

Mean intensive care lenght of stay was observed to be 6.75 days, and it was 9.90 ± 8.25 days in the readmitted group and 6.43 ± 6.64 days in the nonreadmitted group. In the univariate logistic regression analysis, it was shown that an increase in the length of stay in terms of days had an impact on readmission (probability rate 1.052). In their study, Alban et al. found that the intensive care stay duration (4.9 ± 6.7 days) of patients readmitted to the surgical ICU was significantly longer compared with the non-readmitted patients (3.2 ± 6.0 days); moreover, they reported intensive care length of stay as an independent risk factor for readmission [17]. Previous studies have demonstrated increased intensive care length of stay as an independent risk factor for readmission [18,19]. The longer ICU length of stay observed in our study compared with those in other studies can be explained by the fact that our intensive care physician does not make the decision for discharge on his/her own as well as by the low number of beds in the lower-level ICU.

In our ICU, each nurse cares for 2 patients [20]. In the wards, 1 nurse cares for 10 patients. This ratio is worse than the ratio stated in the guidelines (one nurse should care for up to 8 patients) 1NICE-National Institute for Health and Care Excellence (2014) . Safe staffing for nursing in adult inpatient wards in acute hospitals [online]. Website u2962 [accessed 15 July 2014].. It has been emphasized that there are less resources and time in the ward conditions. On the other hand, monitoring frequence of vital functions is less in the service wards, and the ward nurses may monitor vital functions in wider intervals in order to allow the patients to asleep [21]. We believe that readmission to our ICU is increased because of the late detection of patients with worsening status and necessary treatment arrangement not being able to be provided because of these reasons. 

The rate of readmission of patients who were admitted with respiratory failure, COPD, and postcardiac arrest diagnoses were found to be significantly higher. The need for cardiovascular and respiratory support in several patients being monitored in the intensive care is related to the underlying causes of their conditions as well as to the long-term poor health condition [22]. Moreover, our findings for these patient groups were consistent with those of other studies in terms of the long-term health condition. It has been reported that NEWS can predict the risk of sudden cardiac arrest [7].

Accordingly, high NEWS values in patients admitted with cardiac arrest can evidently predict that these patients having an already high risk are likely to worsen.

The power of our study include its prospective design; its inclusion of patients receiving medical and surgical intensive care; conducted in a tertiary ICU; the presence of lower-level ICUs; and the inclusion of possibility of transfer between clinics according to the medical condition of the patients.

Although our number of patients was higher compared to the other prospective studies on this subject, the primary limitation of our study was the inclusion of fewer patients compared to retrospective studies. Second, we were unable to objectively evaluate whether the readmissions were appropriate owing to the observational nature of the study. Owing to legal regulations, a “do not resuscitate” order cannot be given in Turkey, and the admission into intensive care of patients being transferred from palliative care centers is mandatory. We believe that this contributed to our high rate of readmission.

In conclusion, as a result of our study, we found that a NEWS-d value of 7.5 for patients discharged from the ICU showed high sensitivity and specificity for indicating the risk of readmission. NEWS-d score will be a compass for intensivists and its use will cause decreased readmission rates. Therefore, we believe that the NEWS-d scoring system can be a valuable tool in the prevention of readmission of ICU patients.

## Informed consent

Although our study was not an experimental study on humans, informed consent was obtained from the participants.

## Funding

There were no supporting/funding institutions.
